# No evidence of structural abnormality of the substantia nigra in adult attention-deficit/hyperactivity disorder: a pilot cross-sectional cohort study

**DOI:** 10.3389/fpsyt.2024.1395836

**Published:** 2024-05-30

**Authors:** Isabel Friedrich, Daniela von Kuenheim, David Wozniak, Patrick Meyer, Nicole Mauche, Jue Huang, Joseph Classen, Maria Strauss, Jost-Julian Rumpf

**Affiliations:** ^1^ Department of Neurology, University of Leipzig Medical Center, Leipzig, Germany; ^2^ Department of Psychiatry and Psychotherapy, University of Leipzig Medical Center, Leipzig, Germany

**Keywords:** adult attention-deficit/hyperactivity disorder, ADHD, transcranial sonography, echogenic substantia nigra, Parkinson’s disease

## Abstract

**Background:**

Abnormal expansion of the echogenic substantia nigra (SN+) is a common observation in Parkinson’s disease (PD) and considered a potential trait marker within this context. However, SN+ was also frequently detected in children diagnosed with attention-deficit/hyperactivity disorder (ADHD), where it has been discussed as a biomarker of maturational dopaminergic dysfunction. Interestingly, ADHD was recently linked to an elevated risk of PD in epidemiological studies, particularly among individuals treated with psychostimulants. Here, we investigated the potential of SN echogenicity as a disease biomarker in adults with ADHD and its relation to psychostimulant treatment.

**Methods:**

In an exploratory cross-sectional cohort study, we performed transcranial sonography of the SN in 30 adults (mean age 33.3 ± 7.6 years, 19 males/11 females) diagnosed with ADHD according to DSM-V criteria.

**Results and conclusions:**

In this pilot study, we observed no evidence of structural abnormalities of the SN among adults diagnosed with ADHD, thus questioning the potential of SN+ as a biomarker for ADHD in this population. Moreover, we found no evidence of treatment-related SN echogenicity changes that would link therapeutic psychostimulant use to alterations in the structural integrity of the SN.

## Background

Attention-deficit/hyperactivity disorder (ADHD) is a highly prevalent neuropsychiatric disorder in which symptoms first appear in childhood (1). However, many individuals with a history of ADHD in childhood continue to be affected by symptoms of the disease beyond adolescence and require ongoing symptomatic treatment including amphetamine-type psychostimulants (2). Notably, a recent large cohort study reported that individuals with ADHD are at 2.4 times increased risk for movement disorders including Parkinson’s disease (PD) (3). Moreover, the increased risk of developing PD was even six times higher in individuals with ADHD who had received psychostimulants (3). Concerns about a possible causal relationship between the use of amphetamine-type stimulants and increased risk of PD may be supported by epidemiologic studies that reported an increased risk of PD particularly in individuals with illicit methamphetamine use (4–6).

Transcranial sonography (TCS) is a noninvasive imaging technique for detecting abnormalities of midbrain structures including the substantia nigra (SN). In PD, abnormal echogenic SN area expansion (SN+) is considered a trait marker of the disease that can be found in up to 90% of patients (7, 8). SN+ is also detected in about 10–20% of the general population and may, in this context, indicate increased vulnerability or even injury of dopaminergic neurons (9, 10). However, the positive predictive value of SN+ for the development of PD in the general population over the age of 50 years without motor or non-motor signs indicative of PD is low [i.e., ~6% (9)]. Interestingly, abnormal echogenic SN expansion has also been noted in children with ADHD by two independent groups [n=29 children with ADHD, median age 11 years in (11); n=22 children with ADHD, mean age 10.7 years in (12)]. In the context of ADHD in children, SN+ was interpreted as a potential biomarker of dysfunction of the dopaminergic system, possibly caused by developmental delay but unrelated to symptomatic treatment or neurodegeneration (11, 12). However, increased prevalence of SN+ in adults with methamphetamine abuse (13–15) may suggest that amphetamine-type psychostimulants could cause injury to nigral dopaminergic neurons.

In the current study, our primary objective was to investigate, whether the increased frequency of abnormal expansion of the SN observed in children with ADHD persists in adults with ADHD and may, thus, represent a feasible biomarker of the disease. We further investigated, whether the spatial expansion of the echogenic SN may be sensitive to symptomatic ADHD treatment with psychostimulants. The findings will contribute to a thorough comprehension of the echogenic SN within the framework of ADHD and its potential utility as a biomarker for assessing the functionality of the dopaminergic system in this context.

## Methods

We included 30 adults with ADHD according to DSM-V criteria who were recruited from the outpatient clinic at the Department of Psychiatry and Psychotherapy at Leipzig University Medical Center between June 2019 and March 2022 (with interruptions due to the SARS-CoV-2 pandemic). Participants were screened for psychiatric co-morbidity (Structured Clinical Interview for DSM-V: clinician version, SCID-5-CV) (16, 17), completed retrospective symptom acquisition questionnaires for hyperkinetic disorders (Wender-Utah-Rating-Scale short version, WURS-K) (18, 19), Conners’ Adult ADHD Rating Scales (CAARS) (20) and the ADHD Self-Report Scale (ASRS-v1.1) (21). Exclusion criteria encompassed history of structural brain lesions, PD and other basal ganglia or cerebellar disorders, current severe depressive, manic, or psychotic symptoms, acute suicidality, current abuse of illicit drugs and alcohol.

### Transcranial sonography

In each participant, TCS (Acuson S2000, Siemens, Erlangen, Germany) was performed according to consensus criteria (7, 8) by two experienced ultrasound raters separately. The echogenic SN area in each image was determined offline by manually encircling the outline of the echogenic SN. For the purpose of internal quality control, one of the raters (P.M.) was blinded for the study objective and diagnosis of participants. To reduce intra-rater variability mean values of three assessments each of the left and right echogenic SN were entered in the analysis.

### Statistical analysis

Statistical analysis was performed using SigmaPlot (Systat Software, Erkrath, Germany). We applied paired or unpaired student’s t-tests for within- and between-cohort comparisons (or Mann-Whitney rank sum test in case of non-normally distributed values). Pearson correlation was used to test for linear correlations of SN echogenicity with demographic characteristics. The alpha-level was set to 0.05. Since there was no systematic error for echogenic SN assessments between the blinded and unblinded rater (right/left, average; all p≥0.287), we used the assessments of the blinded rater for final analysis.

## Results

**Figure 1:**
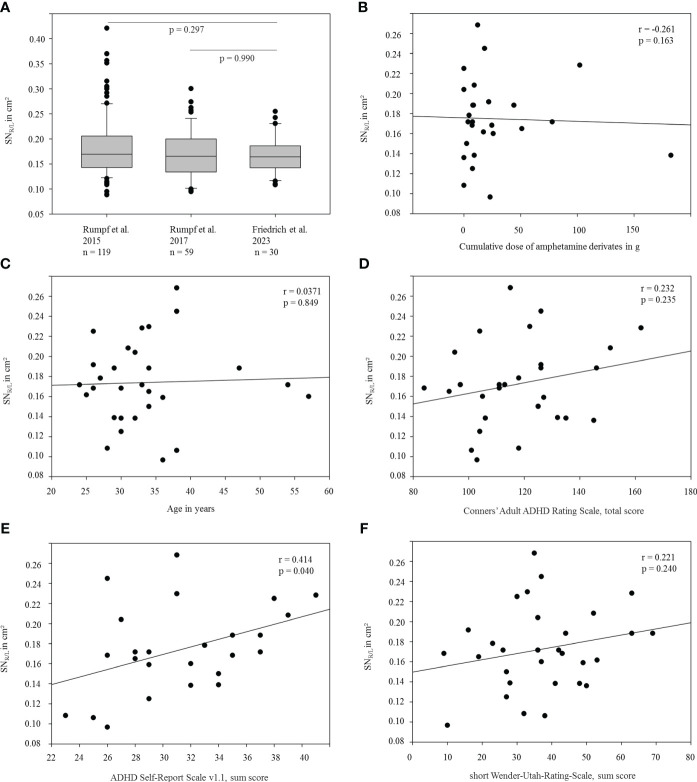
Representation and association of the size of the echogenic SN with selected demographic and clinical variables. **(A)** Average echogenic SN area (SN_R/L_) of the current ADHD cohort compared to independently recruited healthy (control) cohorts from previous studies at our side (13, 23). **(B–F)**: SN_R/L_ area shown as a function of **(B)** the approximated cumulative dose of methylphenidate derivates, **(C)** age, **(D)** scores in Conners’ Adult ADHD Rating Scale, **(E)** scores in ADHD Self-Report Scale v1.1 part B and **(F)** scores in Wender-Utah-Rating-Scale (short version).

For detailed demographic information see **Table 1**. The mean area of right and left echogenic SN (SN_R/L_) amounted to 0.17 ± 0.04 cm² (mean ± standard deviation), and was, thus, well below consensus cut-off values for moderate or marked SN+ [i.e., ≥0.20/≥0.25 cm^2^ (7)] and the cut-off value for SN+ established at our site [≥0.24 cm² (22)], which has also been considered by others to be optimal for distinguishing PD from controls (23). There was no significant difference of echogenic SN expansion in terms of side (left/right, p=0.706), or sex (p=0.697). Furthermore, SN_R/L_ was not significantly different from that of two independent young healthy cohorts from previous studies at our site ( (24): n=116, 51 female, mean age 27.4 ± 4.9, SN_R/L_ 0.18 ± 0.06 cm², p=0.297 (13): n=59, 21 female, mean age 26.9 ± 5.8, SN_R/L_ 0.17 ± 0.05 cm², p=0.990, **Figure 1A**). Prevalence of SN+ (≥0.24 cm^2^ on either side) in the adult ADHD cohort was 13.3% and, thus, did not deviate from the range of SN+ prevalence (~10 – 20%) in several cohorts without PD (9, 13, 24–26). Correlation analysis revealed no relevant association of SN_R/L_ with cumulative intake of methylphenidate derivates (r=-0.261, p=0.163), age (r=0.037, p=0.847), nor with clinical features of ADHD (all p≥0.192, except for self-reported frequency of potentially disease-related symptoms, ASRSv1.1 part B, r=0.414, p=0.040; see **Figures 1B–F**). Participants with a history of methamphetamine abuse (n=5) showed a numerically larger, but not significantly increased, echogenic SN area compared to participants without former methamphetamine use (0.19 ± 0.04 cm² vs. 0.17 ± 0.04 cm², p=0.352).

**Table 1:** Clinical and demographic details of the ADHD cohort (n=30).

Age in years (mean ± SD; range)	33.3 ± 7.6; 24 - 57
Sex (male/female, n)	19/11
Duration of ADHD symptoms in years (mean ± SD; range)	27.3 ± 7.8; 14 – 50
Interval since time of initial ADHD diagnosis in months (mean ± SD; range)	66 ± 106; 1 – 408
Symptomatic pharmacologic therapy for ADHD (%/n)	83.3/25 (%/n)
Duration of symptomatic pharmacologic therapy in months (mean ± SD; range)	31.8 ± 53; 0 – 264
Use of amphetamine derivates (methylphenidate, lisdexamfetamine; %/n) Use of other stimulating agents as monotherapy (atomoxetine; %/n)	76.6/23 6.6/2
Amphetamine derivative dosage equivalents in mg/day (mean ± SD; range)	39.4 ± 12.8; 20 – 50
Cumulative dose of amphetamine derivatives in g (mean ± SD; range)	38.0 ± 64.6; 2.4 – 288.9
Positive illicit drug history (%/n)	60/18
Tetrahydrocannabinol use (%/n)	43.3/13
(Meth)Amphetamine use (%/n)	16.7/5
Diagnosed psychiatric comorbidity (%/n)	70/23
Diagnosed recurrent depressive disorder (%/n)	40/12
WURS-K, sum score (mean ± SD; range)	37.2 ± 14.8; 9 – 69
ASRS-v1.1 part A, sum score, (mean ± SD, range)	16.8 ± 2.1; 13 – 21
ASRS-v1.1 part A, cut-off (mean ± SD; range)	4.9 ± 0.9; 3 – 6
ASRS-v1.1 part B, sum score (mean ± SD; range)	31.4 ± 4.8; 23 – 41
ASRS-v1.1 part B, cut-off (mean ± SD; range)	8.8 ± 2.0; 5 – 12
CAARS, total score (mean ± SD; range)	117.9 ± 18.7; 84 – 162
DSM-G, T-score (mean ± SD; range)	78.6 ± 8.0; 61 – 90
DSM-IA, T-score (mean ± SD; range)	82.8 ± 6.8; 64 – 90
DSM-HYI, T-score (mean ± SD; range)	66.4 ± 11.7; 64 – 90
ADHD-index, T-score (mean ± SD; range)	77.7 ± 7.7; 65 – 90

ADHD, attention-deficit/hyperactivity disorder; ASRS-v1.1, ADHD Self-Report Scale v1.1; CAARS, Conners’ Adult ADHD Rating Scale; DSM (-G, -IA, - HYI), Diagnostic and Statistical Manual of Mental Disorders (-global, -inattention, -hyperactivity/impulsiveness); SD, strandard deviation; WURS-K, short version of the Wender-Utah Rating-Scale.

## Discussion

This exploratory cross-sectional cohort study provided no evidence of abnormal echogenic SN expansion in adults with ADHD according to current consensus cut-offs for SN+ and in comparison to independent control groups in a comparable age range. These findings contrast with previous observations in children with ADHD, in whom a markedly enlarged echogenic SN area was reported and discussed as a potential biomarker for dysfunction of the dopaminergic system (11, 12). Specifically, the observation of a physiological postnatal *decrease* of the echogenic SN area across the first decade of life (27) led to the interpretation that SN+ in children with ADHD may be related to maturational delay of the dopaminergic system (12). However, others (25) reported an almost linear *increase* in echogenic SN area across infancy to adulthood. Given these contradictory observations, it is uncertain whether the hypothesis that SN+ in children with ADHD is linked to developmental delay can be sustained. Our current results suggest that if SN+ in ADHD was indicative of a maturational delay of the dopaminergic system, it does not persist into adulthood despite ongoing symptoms. This is compatible with studies that question dopamine dysregulation as the primary cause for ongoing ADHD symptoms in adults (28). The persisting efficacy of amphetamine-type stimulants in adult ADHD, on the other hand, suggests that dysfunction of the dopaminergic system continues to be clinically relevant. Therefore, if there is dysfunction within the dopaminergic system in adult ADHD, it is not reflected by structural alterations of the SN (at least none that can be detected with TCS). Our observations in adults with ADHD may be supported by a small preliminary study in n=13 young adults with ADHD (mean age 27.3 years), which also found no significant difference in echogenic SN area between ADHD patients and controls (29).

In adults, SN+ is by far most closely associated with PD, in which this trait is present in up to 90% of patients (7). However, SN+ can also be found in 10 – 20% of individuals without PD and may signal increased vulnerability or even injury of dopaminergic neurons (10, 30). Increased prevalence of SN+ in cohorts without Parkinsonian motor symptoms but non-motor prodromal PD symptoms (31–33) appear to suggest that SN+ represents a risk marker of PD. If SN+ may be indicative of an increased PD risk also in ADHD and considering the association of ADHD and PD observed in a recent study (3), one could anticipate an increased frequency of SN+ in adults with ADHD as well. However, in addition to the normal average echogenic SN extension, the prevalence of SN+ in our ADHD cohort fell well within the range observed in healthy controls (9, 13, 24–26). Consequently, if there is a pathophysiological link between ADHD and PD, it is unlikely to be reflected by alterations of the echogenic SN. However, this interpretation must be viewed with caution, particularly in a young cohort without motor or non-motor symptoms indicative of PD, given the low positive predictive value of SN+ with respect to PD risk (i.e., merely 6%) documented in the general population above 50 years of age (9) and the relatively small and young sample (n=30) of the current study. Nevertheless, despite being an exploratory pilot study, the current sample represents the largest cohort examined using TCS within the adult ADHD context. Furthermore, given the substantial effect size of abnormal echogenic SN expansion observed in a previous study in children with ADHD (i.e., 0.92 (12)), and the hypothesis that this putative biomarker for ADHD might persist into adulthood with similar prevalence among individuals exhibiting enduring symptoms, a calculated sample size of n=5 would have sufficed to replicate the earlier findings in pediatric ADHD among adults with ADHD. Even if we assume a lower effect size of at least 0.5, the calculated sample size to detect a significant difference was n=26, thus remaining below our recruited cohort size (n=30). Therefore, we consider it improbable that the negative finding with respect to the primary objective of the study, i.e., whether increased echogenic SN area may be a feasible biomarker of ADHD in adults, is primarily attributable to limitations inherent in smaller sample sizes.

Reports of an increased frequency of SN+ in individuals with methamphetamine abuse (13–15), who were found to be at increased PD risk (4–6), and the observation of a potential methamphetamine dose-dependent expansion of the echogenic SN (13) suggests that the echogenic SN may be sensitive to alterations induced by amphetamine-type stimulants. Here, we found that the expansion of the echogenic SN in adults with ADHD was not associated with the cumulative intake of amphetamine derivates. Again, if there is an intrinsic association between ADHD and PD, the current results do not suggest that this association is related to, or even caused by, psychostimulant-induced injury of SN neurons. Of note, none of our participants received symptomatic treatment with methamphetamine (not approved in Germany). However, consistent with previous research (13–15), we found a numerically (however not statistically significant) larger echogenic SN expansion in participants with former methamphetamine abuse which supports that illicit methamphetamine use may induce alterations of the echogenic SN signature. Nevertheless, this exploratory observation has to be interpreted with caution given the only small number of participants (n=5) who reported a history of illicit methamphetamine use.

## Conclusions

In conclusion, the results of this pilot study suggest that the expansion of the echogenic SN area is, unlike evidence in children with ADHD, not useful as an ADHD biomarker in adults. Moreover, the current results challenge the view that SN+ in ADHD may reflect maturational delay of the dopaminergic system, at least it does not persist in adult ADHD despite persisting symptoms. Our results may further suggest, that if there is an intrinsic link between ADHD and PD, it is likely not reflected by structural alterations of the SN as assessed by TCS. Importantly, we found no evidence of treatment-associated changes in echogenic SN expansion that would link therapeutic use of psychostimulants to alterations of the structural integrity of the SN. Future larger scale studies also utilizing complementary neuroimaging techniques, such as iron-sensitive magnetic resonance imaging, may aid in clarifying whether ADHD or its treatment is associated with structural changes in the SN.

## Data availability statement

The raw data supporting the conclusions of this article will be made available by the authors without undue reservation.

## Ethics statement

The studies involving humans were approved by ethics committee of the University and the Medical Faculty of Leipzig (Reg. no.: 048/19-ek) in accordance with the 1964 Declaration of Helsinki and its lateramendments. The studies were conducted in accordance with the local legislation and institutional requirements. The participants provided their written informed consent to participate in this study.

## Author contributions

IF: Data curation, Formal analysis, Investigation, Project administration, Visualization, Writing – original draft, Writing – review & editing. DvW: Conceptualization, Investigation, Writing – original draft, Writing – review & editing. DW: Investigation, Writing – original draft, Writing – review & editing. PM: Investigation, Methodology, Writing – original draft, Writing – review & editing. NM: Investigation, Writing – original draft, Writing – review & editing. JH: Investigation, Writing – original draft, Writing – review & editing. JC: Writing – original draft, Writing – review & editing. MS: Conceptualization, Writing – original draft, Writing – review & editing. J-JR: Conceptualization, Formal analysis, Supervision, Validation, Writing – original draft, Writing – review & editing.
